# Interfacial Insights into the Efficient Electroconversion of CO_2_ to CO Using Cu-Based Catalysts Supported on Printex L6 Carbon

**DOI:** 10.1021/acsomega.6c02410

**Published:** 2026-08-04

**Authors:** Thalita F. da Silva, Larissa T. Cardoso, Marciélli K. R. de Souza, Julio C. Lourenço, Guilherme V. Fortunato, Marcos R. V. Lanza

**Affiliations:** † São Carlos Institute of Chemistry, University of São Paulo, Trab. São Carlense Avenue, 400, São Carlos, São Paulo 13566-590, Brazil; ‡ Sustainable Energy Materials, Technical University Munich, Campus Straubing, Schulgasse 22, Straubing 94315, Germany

## Abstract

Carbon dioxide (CO_2_) from fossil fuel combustion drives anthropogenic climate change, and electrochemical CO_2_ reduction reaction (CO_2_RR) offers a pathway to convert this waste into valuable chemicals. However, catalytic performance is often discussed in terms of active site density alone, while the role of the local interfacial environment remains insufficiently resolved. Here, a series of Printex L6 carbon-supported copper catalysts (PL6C@Cu_*x*_, where x corresponds to the actual Cu loading wt %) were systematically evaluated for the selective CO_2_-to-CO conversion. Through linear sweep voltammetry analysis, PL6C@Cu_1.04_ was identified as the best-performing catalyst among the catalysts investigated; this catalyst exhibited a marked anodic shift in onset potential (to −0.53 V vs RHE) compared to the bare PL6C and nonoptimized loadings. Rotating ring-disk electrode measurements confirmed CO as the dominant product, detected via oxidation at ∼0.8 V vs RHE. To elucidate the mechanistic origins of the enhancement in catalytic efficiency, the interfacial microenvironment was investigated using a quinone redox probe sensitive to local CO_2_ concentration and adsorption dynamics. The optimized PL6C@Cu_1.04_ catalyst showed an estimated 15-fold increase in the local CO_2_ affinity index compared to the bare PL6C catalyst, associated with a 140 mV positive shift in the half-wave potential of the redox probe. This study offers experimental evidence suggesting that optimizing metal loading not only maximizes active sites but also assists in engineering a CO_2_-enriched microenvironment, which appears crucial for driving efficient and selective electrocatalysis.

## Graphical abstract

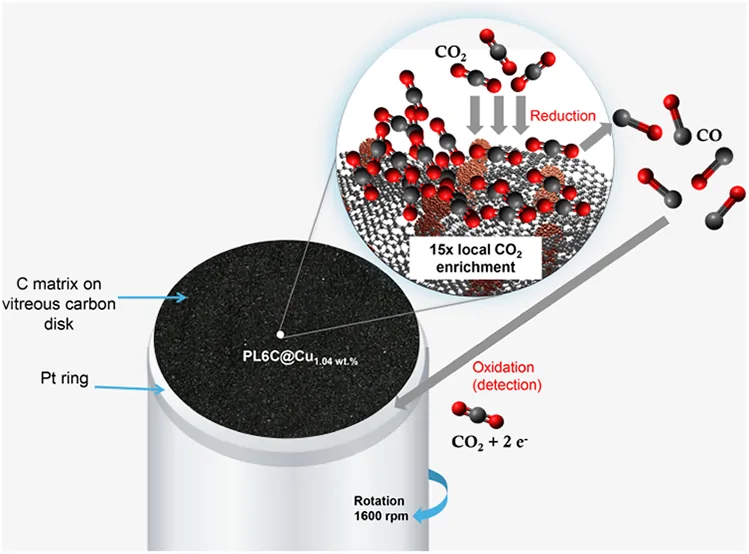

## 1 Introduction

Carbon dioxide (CO_2_) is the main byproduct of fossil fuel combustion and is widely regarded as the primary anthropogenic greenhouse gas that contributes to global climate change.^1–4^ In response to this environmental challenge, the CO_2_ electrochemical reduction reaction (CO_2_RR) has attracted significant attention as a promising strategy for mitigating CO_2_ emissions while converting this abundant molecule into value-added chemicals and fuels, under mild operating conditions. Despite its potential, CO_2_RR remains extremely challenging due to the high thermodynamic stability of the linear CO_2_ molecule and the complexity of its reaction network. The CO_2_RR typically occurs through the formation of the *CO_2_^–^ intermediate, followed by proton-coupled electron transfer reactions, which give rise to *COOH, a key precursor for the formation of carbon monoxide (CO).^5^ The selectivity toward CO and other reduction products is strongly influenced by the catalyst surface, electrolyte composition, mass transport, and the local reaction environment.^6^

The electroconversion of CO_2_ to CO has been widely investigated using a variety of high-performance materials. Noble metals such as gold (Au) and silver (Ag) remain benchmarks due to their low overpotentials, while single-atom catalysts (SACs) and molecular complexes (e.g., phthalocyanines and porphyrins) have recently emerged as powerful tools for achieving near-unity selectivity.^7–10^ However, copper (Cu) has sparked particular interest among researchers due to its moderate binding strength toward carbon-containing intermediates.^11,12^ While bulk copper surfaces are often associated with multicarbon product formation, recent studies have demonstrated that highly dispersed Cu species at low loadings can favor selective CO production by modifying the interfacial reaction environment and suppressing the competing hydrogen evolution reaction (HER).^13^ In this context, interfacial properties such as local pH, electric double-layer structure, and charge-transfer kinetics play a decisive role in governing catalytic activity and selectivity.

Carbon-based supports are particularly effective in tuning these interfacial properties. While carbon black materials like Vulcan XC-72 are ubiquitous in electrocatalysis, there is a compelling need to explore alternative carbon supports that may offer superior structural or electronic properties. In recent years, Printex L6 carbon (PL6C), a commercial conductive carbon black, has gained traction in various electrochemical domains, though it remains under-utilized in CO_2_RR. Characterized by a high specific surface area (∼270 m^2^ g^–1^) and excellent electrical conductivity, PL6C has demonstrated remarkable performance when applied in the electrochemical generation of hydrogen peroxide (H_2_O_2_) through oxygen reduction reaction (ORR). Studies have shown that PL6C favors the 2-electron pathway more efficiently than Vulcan XC-72 primarily due to its distinct surface oxygen functional groups and structural properties.^14,15^

Carbon supports generally exhibit edge defects with the presence of oxygen and/or nitrogen containing groups that can modify the electronic structure of the metallic active sites and stabilize key reaction intermediates, thereby enhancing CO_2_RR performance.^16^ More recently, advances in active-site engineering have demonstrated that Cu dispersion plays a decisive role in CO selectivity. For instance, Pérez-Rodríguez et al. (2024) systematically varied the metal loading and Cu precursor in the synthesis of Cu–N–C catalysts. Using rotating ring-disk electrode (RRDE) analysis, the authors demonstrated high CO selectivity at low overpotentials.^17^ These findings show that establishing precise control over the amount and nature of Cu sites can steer the CO_2_RR reaction pathway. Despite these advances, there remains a lack of comprehensive understanding regarding how the incorporation of Cu into commercial carbonaceous matrices, characterized by the presence of multiple types of active sites, affects CO generation under well-defined mass transport conditions.

Advanced electroanalytical techniques are essential for elucidating such interfacial effects. The rotating ring-disk electrode (RRDE) technique provides a powerful platform for probing reaction pathways under well-defined mass transport conditions, enabling real-time detection of reaction products and intermediates. Although the RRDE technique has been extensively employed for reactions such as oxygen reduction and hydrogen evolution, its application to CO_2_RR remains comparatively limited. Zhang et al. (2020) established quantitative protocols for the detection of CO, H_2_, and formate using different metallic catalysts, where they found that the RRDE technique drastically reduces the time between product generation and analysis compared to ex situ techniques such as gas chromatography or nuclear magnetic resonance (NMR).^18^ Additional studies have shown that hydrodynamics strongly influence the competition between CO_2_RR and HER, as demonstrated by Goyal et al. (2022; 2020), who correlated electrode rotation with changes in selectivity on Au surfaces under controlled mass-transport conditions.^19,20^

Taking the considerations above into account, the present study reports the synthesis and characterization of copper nanoparticles supported on commercial Printex L6 carbon (PL6C@Cu_*x*_) applied as a novel electrocatalyst for CO_2_ reduction. While copper is widely recognized for its ability to promote C–C coupling, the use of advanced carbon supports like Printex L6 allows for the modulation of the interfacial microenvironment, potentially shifting selectivity toward specific intermediates by regulating local reactant concentration and pH. By combining structural and electrochemical characterization with RRDE measurements, impedance spectroscopy, capacitance analysis, and local pH probing using benzoquinone, this study systematically elucidates how copper dispersion and interfacial properties govern CO_2_RR activity and selectivity. The findings of the study highlight the critical role played by the catalyst-electrolyte interface in enabling efficient CO formation and point to the usefulness of RRDE as a diagnostic tool for advanced analysis of CO_2_RR electrocatalysts.

## 2 Materials and Methods

### 2.1 Reagents

Copper chloride (CuCl_2_·2H_2_O, Sigma-Aldrich ≥ 99.0%), urea (NH_2_CONH_2_, Sigma-Aldrich ≥ 99.9%), Nafion solution (Sigma-Aldrich 5 wt %), and potassium bicarbonate (KHCO_3_, Sigma-Aldrich ≥ 99.7%) were used as received. Printex PL6C was purchased from Orion S.A. Nitrogen (N_2_ ≥ 99.0%) and carbon dioxide (CO_2_ ≥ 99.0%) gases were acquired from Messer Gases for Life. Ultrapure water (Milli-Q, 18.2 MΩ cm^–1^) was used to prepare the electrolyte solutions for the synthesis processes and to wash the glassware.

### 2.2 Preparing the PL6C@Cu_x_ Electrocatalyst

PL6C-based electrocatalysts decorated with Cu were synthesized via the hydrothermal method. Prior to the synthesis, PL6C was subjected to a leaching process using 40 mg of carbon in 30 mL of a solution containing H_2_SO_4_ and HNO_3_, both at 0.5 mol L^–1^.^21^ At first, 50 mg of PL6C was dispersed in 75 mL of deionized water in a beaker, which was followed by the sequential addition of 25 mg, 50 mg, 75 mg, and 150 mg of CuCl_2_. Urea was then added in a molar ratio of 100:1 (urea:CuCl_2_), and the mixture was stirred at 45 °C for 30 min. The resulting solution was transferred to a Teflon-lined stainless steel hydrothermal reactor and maintained at 150 °C for 16 h. After cooling to room temperature, the product was washed four times with ultrapure water via centrifugation and dried at 40 °C for 24 h in a drying oven. Finally, the material was calcined at 230 °C under a N_2_ atmosphere for 2 h to reduce Cu.^22^ For comparison purposes, an unmodified PL6C catalyst was prepared by subjecting bare PL6C to the same procedure described above but without CuCl_2_. The samples were named based on their experimentally determined copper content, as quantified via polarographic analysis (see Supporting Information). The materials were named PL6C (bare PL6C) and PL6C@Cu_*x*_, where x corresponds to the actual Cu loading (wt %).

### 2.3 Physicochemical Characterization

The surface morphology of the catalysts was analyzed by scanning electron microscopy (SEM) using a Japan Electron Optics Ltd. (JEOL) JSM 7200F microscope. The morphology and structure of the catalysts were investigated using a Thermo Fisher Talos F200X transmission electron microscope (TEM) equipped with a field emission gun (FEG), operating at 200 kV. High-resolution TEM (HRTEM) and scanning transmission electron microscopy (STEM) images were acquired using a high-angle annular dark-field (HAADF) detector to evaluate nanoparticle dispersion and lattice fringes. Elemental mapping and composition analysis were performed via energy-dispersive X-ray spectroscopy (EDS). Particle size distribution histograms were generated using the ImageJ software. The X-ray diffraction (XRD) analysis was performed in the 2θ range of 10° to 90° using X-ray diffractometer (Bruker, D8 Advance) equipped with Cu Kα radiation (λ = 1.542 Å). Raman spectra were acquired using a Horiba Raman spectrophotometer coupled to an AFM (model LabRam HR Evolution) at room temperature, over the spectral range of 4000–50 cm^–1^. The copper concentration in PL6C was determined by polarography; further details are described in the Supporting Information.

Electrochemical impedance spectroscopy (EIS) experiments and the determination of the double-layer capacitance (C_dl_) were performed using a PGSTAT-128N bipotentiostat/galvanostat (equipped with the FRA2.X module) from Autolab (Metrohm). EIS measurements were conducted over the frequency range of 0.01 to 1.0 MHz at the open-circuit potential (OCP), where no faradaic currents occur, with a sinusoidal perturbation of 0.01 V_rms_.

The electrochemically active surface area (ECSA) was determined using C_dl_.^23^ For this determination analysis, cyclic voltammograms (CVs) were recorded in the OCP region within a potential window of 0.05 V. The CV analyses were performed at scan rates ranging from 5 to 80 mV s^–1^, starting from the most positive to the most negative potential, with a stabilization time of 20 s at each potential limit. The C_dl_ value was then determined as per eq 1

1 Cdl=(Δi2)ν=((ia−ic)2)ν

Where *i*_*a*_ and *i*_*c*_ are the anodic and cathodic currents at OCP, respectively, and v is the potential scan rate. The ECSA was estimated by dividing the C_dl_ value by the specific capacitance (C_s_), with Cs assumed to be 0.035 mF cm^–2^.^24,25^

The copper-specific surface area (*SS*A_Cu_)^26^ was estimated based on the difference between the C_dl_ of the PL6C@Cu_*x*_ composites and the bare PL6C support. The *SS*A_Cu_ (expressed in m^2^ g_Cu_^–1^) was then calculated by normalizing this area to the actual mass of copper deposited on the electrode, as described by the following eqs 2 and 3:

2 $${\mathrm{ECSA}}_{\mathrm{Cu}}\left({\mathrm{cm}}^{2}\right)=\frac{C_{\mathrm{dl},\mathrm{sample}}{\mathrm{-C}}_{\mathrm{dl bare}}}{C_{s}}{\times A}_{\mathrm{geom}}$$

3 $${\mathrm{SSA}}_{\mathrm{Cu}}\left(m^{2}{\mathrm{gCu}}^{-1}\right)=\frac{ECSA_{Cu}}{m_{Cu}}\times 10^{-4}$$

where A_geom_ is the geometric area of the electrode and m_Cu_ is the mass of copper calculated from the 80 μg cm^–2^ total catalyst loading and the respective weight percentages.

### 2.4 Electrochemical Measurements

The electrochemical activity of the catalysts was evaluated in an electrochemical cell equipped with a rotating ring-disk electrode (RRDE, E7R9 ThinGap Fixed-Disk, Pine Research), which consisted of a glassy carbon disk (geometric area of 0.2475 cm^2^) and a Pt ring (geometric area of 0.1866 cm^2^). The collection efficiency of the RRDE electrode was 37%, as specified by the manufacturer. A graphite carbon rod was used as the counter electrode, while an Ag/AgCl (3 mol L^–1^ KCl) electrode was used as the reference electrode. Prior to each analysis, the RRDE was polished using a microcloth (Buehler) and alumina suspensions of 0.1 and 0.05 μm (Buehler), followed by sonication in isopropanol and deionized water for 3 min each to remove impurities. After this procedure, the Pt ring electrode was electrochemically polished by cycling the potential between −0.2 and 1.1 V vs Ag/AgCl (3 mol L^–1^ KCl) at 900 mV s^–1^ for 200 cycles in 0.5 mol L^–1^ H_2_SO_4_ under N_2_ purge, until a stable Pt cyclic voltammogram was observed.

The catalytic microfilm was prepared by dispersing 1 mg of the catalyst in 1 mL of isopropanol, forming an ink suspension. An appropriate volume of this ink was deposited onto the RRDE disk using the drop-casting approach until a loading of approximately 80 μg cm^–2^ was obtained.^27^ After drying, 3 μL of a 0.05% Nafion solution in isopropanol was added to the microfilm to minimize potential catalyst leaching.

The analysis was performed using an Autolab PGSTAT-128N bipotentiostat/galvanostat paired with an MSR rotator with OD PEEK shaft (AFMSRCE, Pine Research). The CO_2_RR was investigated using linear sweep voltammetry (LSV). For the detection of H_2_, CO, and HCOOH as byproducts, chronoamperometry analysis was conducted on the disk electrode at specific reduction potentials, while cyclic voltammetry was simultaneously performed on the ring electrode, which acted as a detector, to monitor the oxidation of the generated byproducts.^19,28^ The apparent partial current density for CO (*j*_*CO*_) was estimated from the ring oxidation current (*I*_*ring*_) using the collection efficiency (N = 0.37) according to eq 4:

4 $$j_{\mathrm{CO}}=\frac{I_{\mathrm{ring}}}{N\cdot A_{\mathrm{disk}}}$$

Where A_disk_ is the geometric area of the disk electrode (0.2475 cm^2^). This approach allows for the quantification of the CO produced at the disk and immediately detected at the ring, providing a localized measure of selectivity.

The measurements were conducted at a rotation speed of 1600 rpm to ensure efficient mass transport. The electrolyte employed consisted of 0.1 mol L^–1^ KHCO_3_ solution, saturated with N_2_ or CO_2_ for 30 min before the measurements. The reported currents were normalized to the geometric area of the working electrode. The detection limit for CO oxidation at the ring was established based on the baseline stability in N_2_-saturated electrolyte. Given the high signal-to-noise ratio observed at the onset of CO_2_ reduction, the detection threshold was determined to be significantly lower than the recorded catalytic currents. This qualitative validation ensures that the potential-dependent onset potentials reported for the synthesized catalysts are not influenced by background fluctuations or instrumental noise.

Unless otherwise stated, all potential values are referenced to the reversible hydrogen electrode (RHE), with conversions performed according to the Nernst equation (eq 5).

5 $$E_{\mathrm{RHE}}{=E}_{\mathrm{Ag}/\mathrm{AgCl}}+\left(0.059\times \mathrm{pH}\right){+E}_{\mathrm{Ag}/\mathrm{AgCl}}^{{}^{\circ}}$$

Where E_Ag/AgCl_ represents the standard thermodynamic reduction potential (0.210 V) of Ag/AgCl in 3 mol L^–1^ KCl. The pH of the electrolyte saturated with CO_2_ was 6.8, while that of the electrolyte saturated with N_2_ was 8.4. In addition to the conversion to RHE, an 85% ohmic drop compensation was applied. The uncompensated resistance (R_u_) was determined by electrochemical impedance spectroscopy (EIS), which yielded a value of 60 Ω.^29^ The potential was manually compensated as per eq 6

6 $$E_{i\mathrm{R corrected}}=\mathrm{E-}iR_{u}f$$

Where E represents the measured potential converted to RHE, *i* is the recorded current, and *f* is the compensation factor set to 0.85. All experiments were conducted in triplicate at room temperature.

## 3 Results and Discussion

### 3.1 Morphological, Structural, and Compositional Analyses

A total of five materials, including commercial Printex L6 carbon (PL6C) and copper-decorated PL6C composites, were investigated. A bare PL6C sample was subjected to the same hydrothermal treatment as the Cu-containing PL6C samples for fair and consistent comparison purposes. A schematic illustration of the hydrothermal process can be found in Figure 1. The copper content of the treated samples was quantitatively determined by differential pulse polarography using a static mercury drop electrode (SMDE). A well-defined reduction peak at −0.01 V versus Ag/AgCl, attributed to the reduction of Cu^2+^ to metallic Cu^0^, was observed for all the Cu-containing PL6C samples. Copper loadings were calculated using the standard addition method, where atomic copper contents of 0.11%, 0.48%, 1.04%, and 1.24% were recorded for samples prepared with 25, 50, 75, and 150 mg of CuCl_2_, respectively.

**Figure 1:**
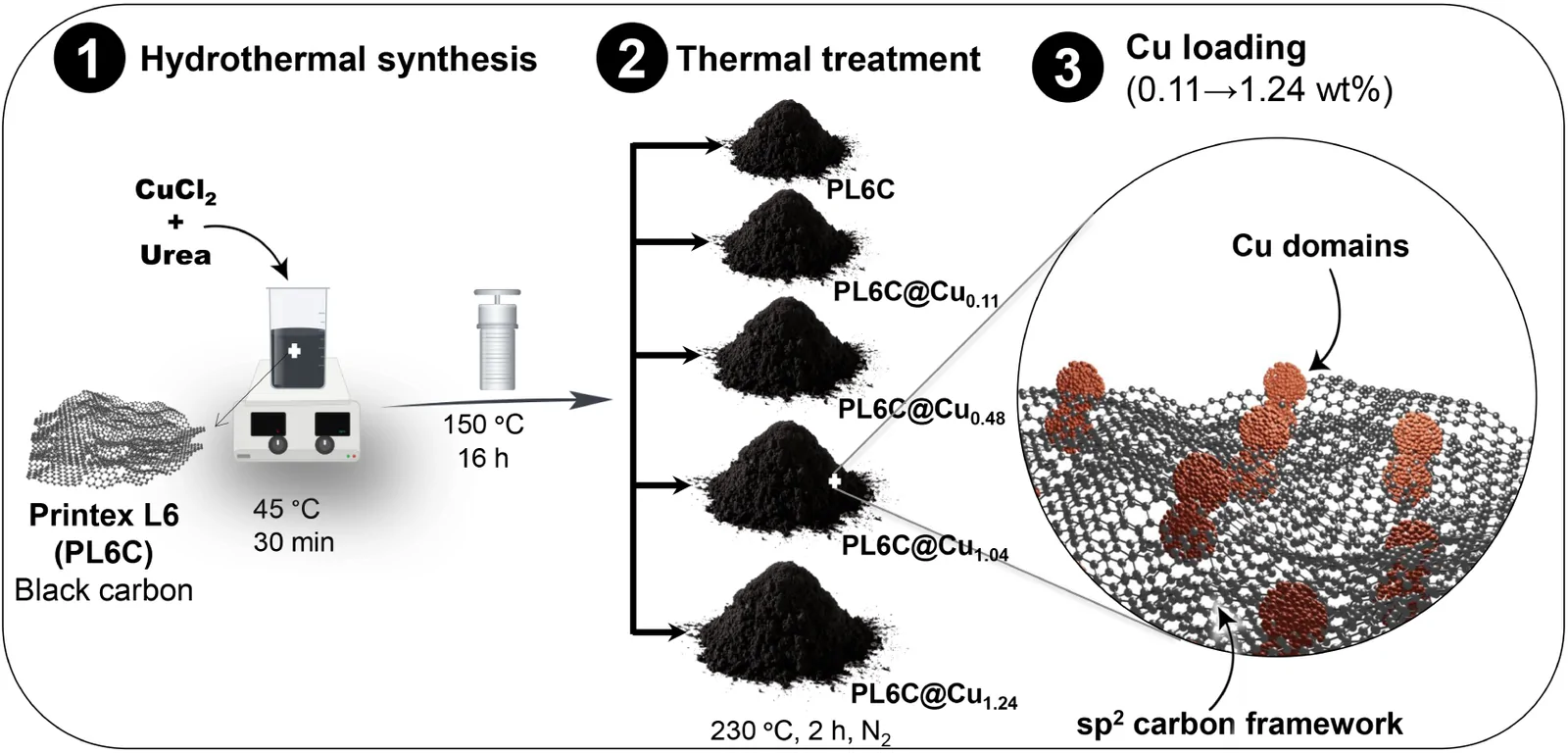
Schematic illustration of the steps involving the preparation of the PL6C@Cu*_x_*.

As observed from the scanning electron microscopy (SEM) images shown in Figure 2, the carbon aggregates exhibited a pseudospherical morphology with a homogeneous distribution of the formed clusters, which is a typical feature of Printex L6 carbon black. Figure 2f and g show the EDS mapping of the PL6C@Cu_1.04_ and PL6C@Cu_1.24_ samples, respectively, where a uniform distribution of Cu throughout the carbonaceous PL6C structure can be clearly observed. Figures S1a–e (see Supporting Information) show the particle size distribution histograms of the copper catalysts supported on the carbon. The bare PL6C catalyst exhibited an average particle size of 45.41 ± 0.22 nm, which is consistent with the value reported by Cordeiro-Junior et al. (2022) for this type of amorphous carbon materials (44.6 nm).^30^ Upon the incorporation of copper, there was a progressive reduction in the particle size of the PL6C aggregates. This decrease, reaching approximately 25.74% for the PL6C@Cu_1.24_ sample, is likely associated with the partial disaggregation of the carbon secondary structures during the hydrothermal synthesis and sonication steps.

**Figure 2:**
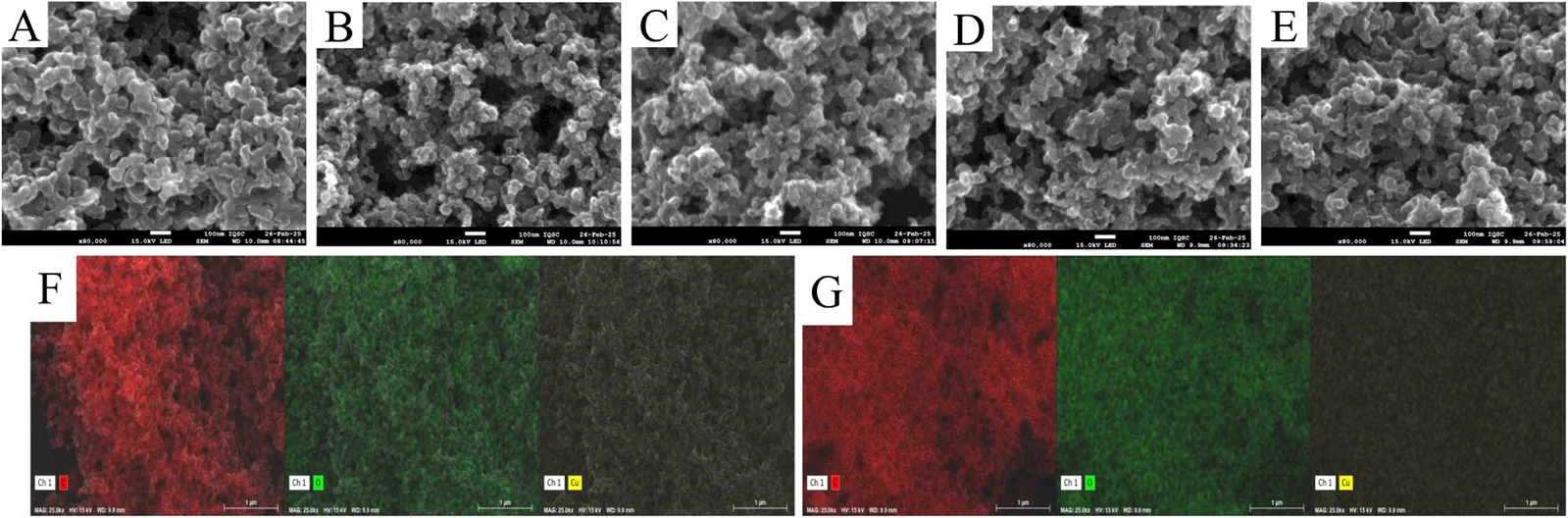
Surface morphology of the synthesized materials. (a) PL6C, (b) PL6C@Cu_0.11_, (c) PL6C@Cu_0.48_, (d) PL6C@Cu_1.04_, (e) PL6C@Cu_1.24_. EDS mapping of the Cu element for (f) PL6C@Cu_1.04_ and (g) PL6C@Cu1.24.

HR-TEM and STEM-HAADF analyses were performed to investigate the morphology and distribution of the Cu nanoparticles. As shown in the STEM-HAADF images (Figure 3a–d), despite the low density due to the low metal loading, the Cu nanoparticles are homogeneously dispersed across the PL6C support. The average size of the particles present in the micrographs exhibited a nonlinear trend: 8.40 nm (PL6C@Cu_0.11_), 10.22 nm (PL6C@Cu_0.48_), 8.76 nm for the optimized catalyst (PL6C@Cu_1.04_), and 9.00 nm (PL6C@Cu_1.24_). As reported by Reske et al. (2014), reducing Cu particle size below 15 nm, particularly under 5 nm, is associated with changes in catalytic activity and selectivity toward H_2_ and CO.^31^ This behavior is often attributed to the higher density of structurally unsaturated active sites, such as edges and corners, in smaller particles.

**Figure 3:**
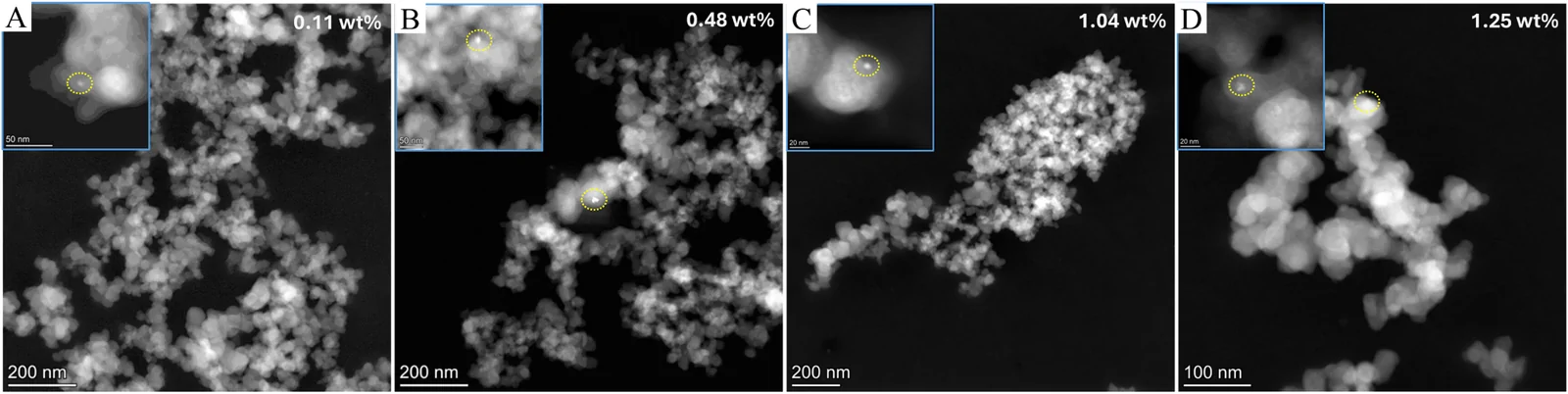
STEM-HAADF images of catalysts with different Cu loadings: (a) PL6C@Cu_0.11_, (b) PL6C@Cu_0.48_, (c) PL6C@Cu_1.04_, and (d) PL6C@Cu_1.24_. The circled bright spots represent the Cu nanoparticles dispersed on the PL6C support.

In the PL6C@Cu_1.24_ catalyst, the formation of copper nanoparticle clusters was observed (Figure S2a), with EDS mapping (Figure S2b) confirming a homogeneous distribution of copper within these regions. HR-TEM analysis (Figure S2c) revealed a lattice *d*-spacing of 0.25 nm, characteristic of the Cu_2_O (111) planes, which was further supported by nanodiffraction (Figure S2d). The presence of this oxide phase is consistent with the natural surface oxidation of small-sized copper nanoparticles upon environmental exposure.^32,33^

X-ray diffraction (XRD) analysis was carried out in order to investigate the crystallographic phase composition of the PL6C@Cu_*x*_ catalysts, in both bare and modified forms, see Figure 4a. A broad and intense peak was observed at 24.53°, along with a second broad but less intense peak at 43.00°, which can be indexed to the (002) and (100) planes of graphitic-like carbon, respectively.^14,34^ These peaks are associated with the stacking of carbon layers and the in-plane ordering of the carbon framework, and are consistent with the standard XRD profile of carbon black (PDF 73-2096). Characteristic peaks of metallic Cu (PDF 65-9743), CuO, and Cu_2_O oxides were not observed in the diffraction patterns. Thus, it can be assumed that the synthesized material exhibits amorphous characteristics, or that the amount and/or size of copper particles is insufficient to generate a detectable signal.^35^ The absence of diffraction peaks related to metallic Cu or copper oxides suggests that copper species are highly dispersed, amorphous, or present as ultrasmall nanoparticles below the detection limit of XRD. For carbon black-supported catalysts, the high background from the amorphous support further limits XRD sensitivity to low copper loadings.

**Figure 4:**
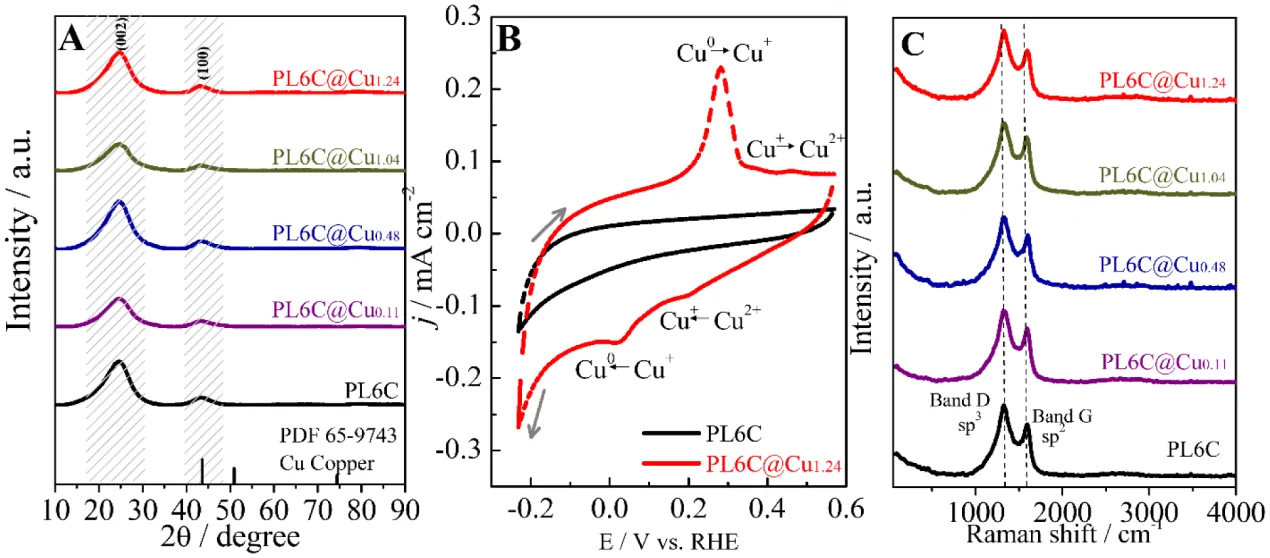
Physicochemical characterization of the synthesized materials. (a) XRD patterns of the materials. (b) Cyclic voltammograms obtained for PL6C and PL6C@Cu_1.24_ based on the application of 0.1 mol·L^–1^ H_2_SO_4_ as supporting electrolyte, in the potential range of 0.56 V to −0.23 V vs RHE, with scan rate of 50 mV·s^–1^. (c) Raman spectra of the materials.

A representative cyclic voltammogram of a surface containing Cu nanoparticles is shown in Figure 4b; it displays an anodic peak at 0.25 V vs RHE (E_pa_) and a cathodic peak at −0.15 V vs RHE (E_pc_), which are attributed to the Cu^0^/Cu^+^ redox process in the PL6C@Cu_1.24_. A second, less intense anodic peak is observed at 0.45 V vs RHE (E_pa_), along with a cathodic peak at −0.08 V vs RHE (E_pc_), associated with the Cu^+^/Cu^2+^ redox couple. As expected, the bare PL6C sample showed no redox peaks. Similar voltammetric profiles with the same redox features are observed for the PL6C@Cu_0.11_, PL6C@Cu_0.48_, and PL6C@Cu_1.04_, Figure S3 (see Supporting Information). The observation of well-defined Cu^0^/Cu^+^ and Cu^+^/Cu^2+^ redox couples confirms the presence of electrochemically accessible copper species at the catalyst-electrolyte interface. The coexistence of multiple copper oxidation states points to a dynamic copper surface under electrochemical conditions, which has been widely associated with enhanced CO_2_RR activity, with the stabilization of key reaction intermediates.

Raman spectroscopy analysis was carried out in order to assess the degree of aromaticity in the samples. Figure 4c shows the Raman spectra obtained for the synthesized catalysts. All the materials investigated exhibited the two characteristic bands of amorphous carbon: the D band, centered at 1323 cm^–1^, and the G band, at 1587 cm^–1^. The D and G bands are associated with the physicochemical properties of carbonaceous materials, depending on their relative intensities. The D band is attributed to structural defects in the graphitic lattice, such as edges, vacancies, and oxygen-containing functional groups, and is commonly linked to sp^3^-type bonding. In contrast, the G band corresponds to the vibrational modes of sp^2^-hybridized carbon atoms in a graphitic network, which indicate the degree of structural order.^36^ To evaluate the aromatic character, the D-band to G-band intensity ratio (I_D_/I_G_) was calculated for all the materials investigated. Table S1 provides an outline of the values obtained, where the catalysts can be found to reflect the following order of increasing structural disorder: PL6C@Cu_1.24_ < PL6C@Cu_0.48_ < PL6C@Cu_0.11_ < PL6C < PL6C@Cu_1.04_.

### 3.2 Interfacial Electrochemical Properties under N_2_ and CO_2_

To elucidate the kinetics of charge transfer and mass transport at the electrode–electrolyte interface, electrochemical impedance spectroscopy (EIS) analysis was performed.^37^ Figure 5a shows the Nyquist plots obtained for the investigated catalysts based on the application of 0.1 mol·L^–1^ KHCO_3_ solution, saturated with CO_2_, as supporting electrolyte. The high-frequency region of the Nyquist plot is shown in Figure S4 (see Supporting Information). At high frequencies, typically above 1000 Hz, the electrochemical response can be found to be predominantly resistive, while at intermediate and low frequencies, a capacitive behavior is observed. This effect is better visualized in the Bode plot shown in Figure 5b, where the transition point between these regimes is determined. At low frequencies, there is a predominance of diffusive effects, where mass transport becomes the limiting factor. At high frequencies, a slope close to zero indicates purely resistive behavior. These effects are consistently observed in the synthesized materials. Figure 6c shows the fitted Randles equivalent circuits for the different materials. From these fittings, it was possible to determine the values of solution resistance (R_s_), double-layer capacitance (C_dl_), charge transfer resistance (Rct), capacitance associated with mass transport (C_mt_), and resistance to mass transport (R_mt_).

**Figure 5:**
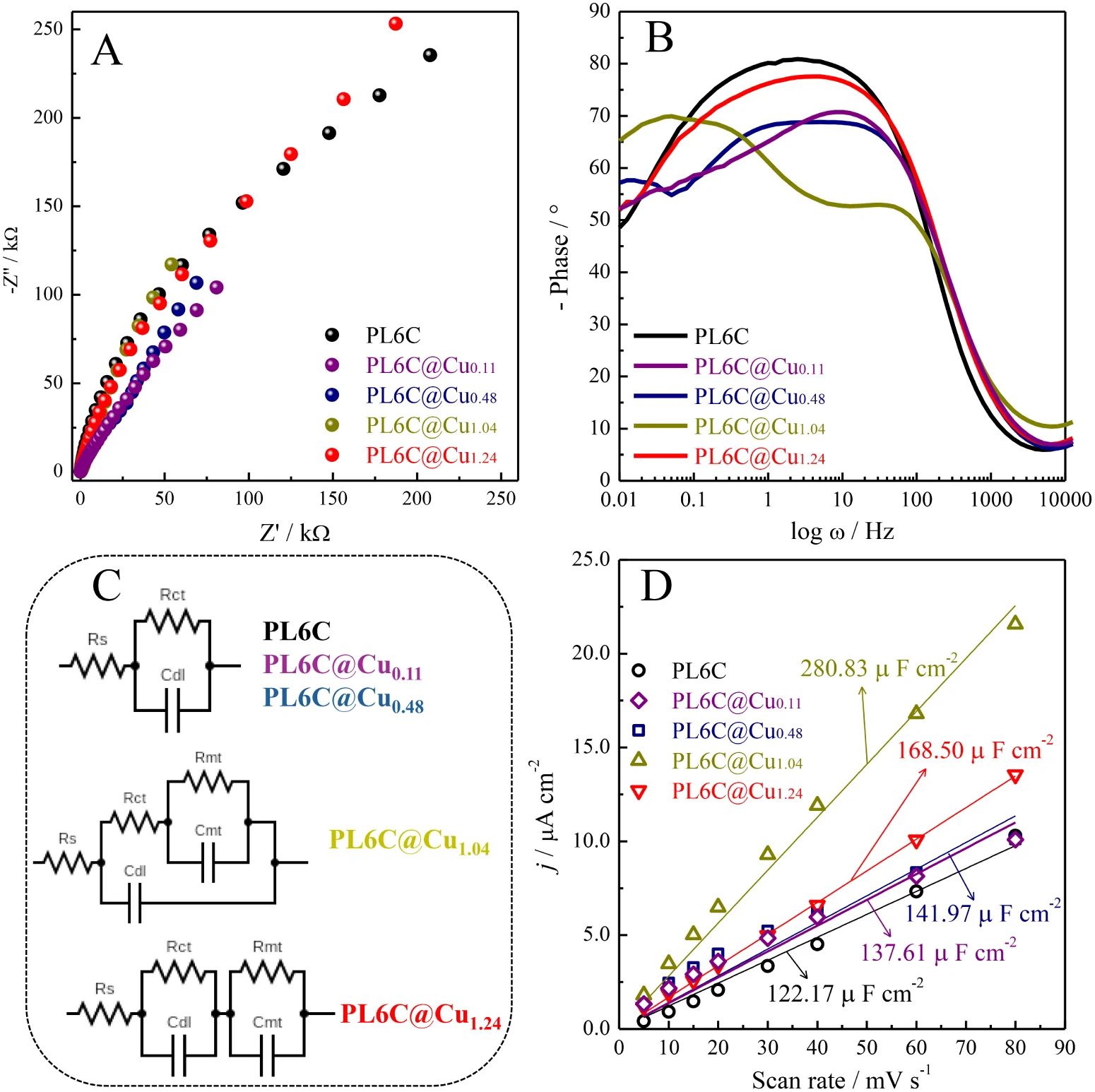
Electrochemical impedance spectroscopy (EIS) results and interfacial capacitance of the catalysts. (a) Nyquist plot. (b) Bode plot, (c) Randles circuit for the obtained impedance data, and (d) Interfacial capacitance as a function of potential. All measurements were performed near the open-circuit potential (OCP) using CO_2_-saturated 0.1 mol·L^–1^ KHCO_3_ as supporting electrolyte.

**Figure 6:**
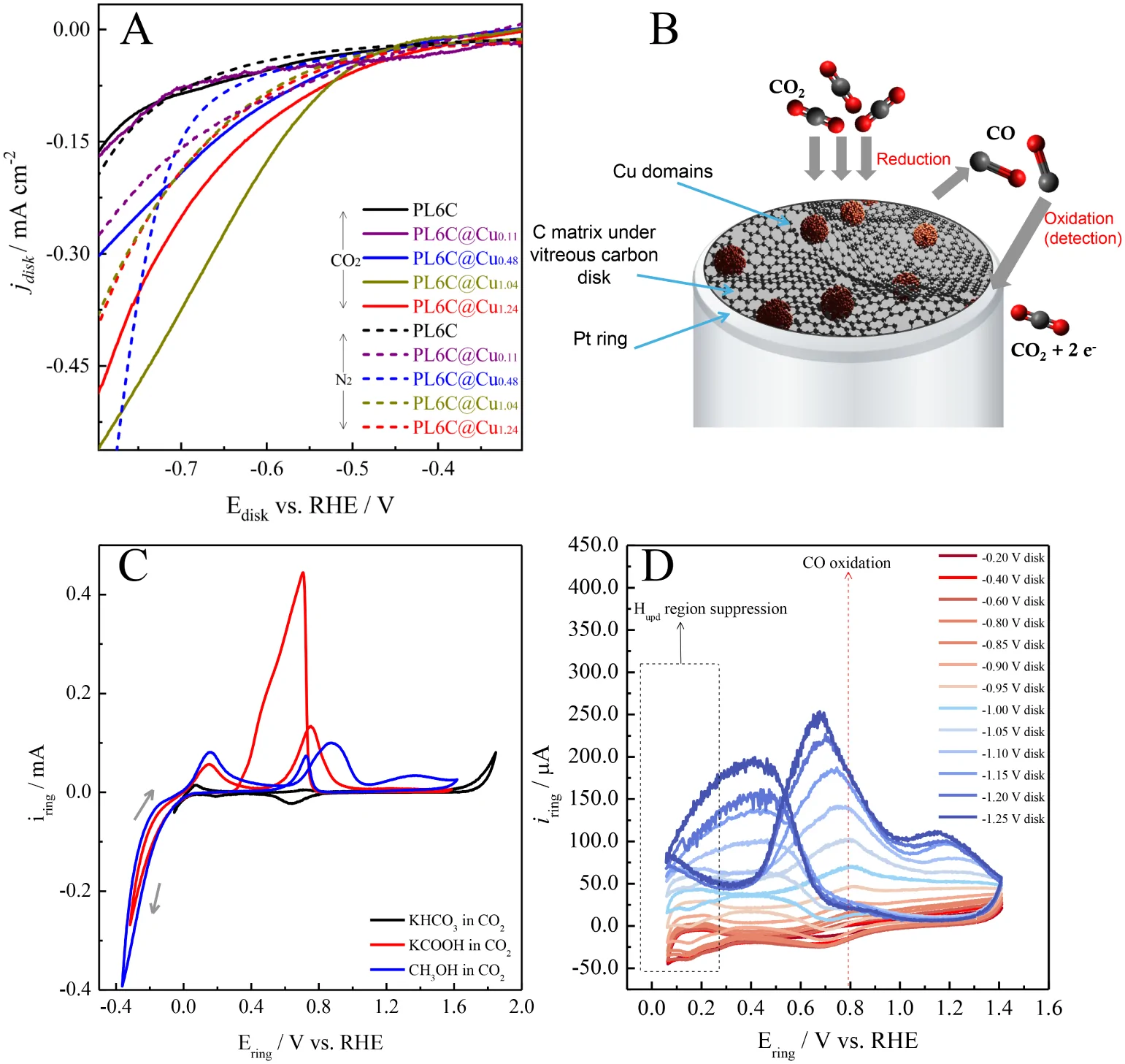
RRDE-based electrochemical analysis of CO_2_ reduction based on the application of the PL6C@Cux catalysts. (a) Linear sweep voltammetry (LSV) curves recorded in N_2_- and CO_2_-saturated 0.1 mol·L^–1^ KHCO_3_ at 1600 rpm, with scan rate of 5 mV·s^–1^. (b) Schematic representation of the RRDE, illustrating CO_2_ reduction on the disk and oxidation of soluble products on the Pt ring. (c) Cyclic voltammograms showing the oxidation behavior of model CO_2_RR products (KHCO_3_, HCOOK, and CH_3_OH) on the platinum ring electrode. (d) Cyclic voltammograms recorded on the Pt ring under different fixed potentials applied to the glassy carbon disk electrode modified with PL6C@Cu_1.04_.

A thorough analysis of the transfer function brought to light parameters related to reaction kinetics and mass transport, as presented in Table 1. These data were acquired from experiments conducted in H_2_SO_4_ saturated with N_2_ and in KHCO_3_ saturated with either CO_2_ or N_2_. A clear trend in electrochemical behavior is observed in the transition from an N_2_-saturated (inert) electrolyte to a CO_2_-saturated (reactive) electrolyte, particularly for the PL6C@Cu_1.04_ composite. In N_2_-saturated KHCO_3_, the Rct for the PL6C@Cu_1.04_ was 12.4 kΩ. Upon the introduction of CO_2_, this value decreased dramatically to 0.8 kΩ. This approximately 15-fold decrease in resistance indicates enhanced electrocatalytic activity of the Cu nanoparticles toward CO_2_ reduction. In contrast, the bare PL6C catalyst exhibited a pronounced increase in R_ct_ under CO_2_ atmosphere (from 55.4 to 318.4 kΩ), indicating that the carbon surface is essentially inactive for the electrochemical reduction of CO_2_. Furthermore, the emergence of additional elements, namely an R_mt_ of 7.9 kΩ and a C_mt_ of 32.02 μF, exclusively for the PL6C@Cu_1.04_ sample under CO_2_, suggests a shift in the rate-determining step. The surface reaction kinetics become sufficiently fast such that CO_2_ diffusion through the porous Printex L6 structure and local pH gradients become kinetically relevant, a phenomenon consistent with boundary-layer models described by Gupta et al. (2006).^38^ Additionally, the pronounced decrease in C_dl_ recorded by the PL6C@Cu_1.04_ under CO_2_ atmosphere (8.52 μF) compared to N_2_, this effect may arise from changes in the ionic composition of the electrolyte (HCO_3_^–^/CO_3_^2–^ formation), adsorption of carbonate species, or modifications in the surface oxidation state of Cu.

**Table 1:** Parameters Related to Reaction Kinetics and Mass Transport Recorded for the Synthesized Materials

**Catalyst**	Experimental condition^a^	OCP/V	**R**_**s**_ **/Ω**	**C**_**dl**_ **/μF**	**R**_**ct**_ **/kΩ**	**C**_**mt**_ **/μF**	**R**_**mt**_ **/kΩ**	**χ** ^**2**^
PL6C	H_2_SO_4_/N_2_	0.291	17.3	33.72	545.0	-	-	0.2096
	KHCO_3_/N_2_	–0.057	60.1	157.42	55.4	-	-	0.0687
	KHCO_3_/CO_2_	0.106	67.4	26.75	318.4	-	-	0.4457
PL6C@Cu_0.11_	H_2_SO_4_/N_2_	0.402	16.4	94.69	144.0	-	-	0.2648
	KHCO_3_/N_2_	–0.075	56.3	49.11	117.5	-	-	0.2987
	KHCO_3_/CO_2_	0.140	62.3	56.39	110.8	-	-	0.0280
PL6C@Cu_0.48_	H_2_SO_4_/N_2_	0.387	15.7	67.43	244.0	-	-	0.4159
	KHCO_3_/N_2_	–0.039	60.7	30.17	314.6	-	-	0.0571
	KHCO_3_/CO_2_	0.245	61.2	95.75	285.9	-	-	0.2961
PL6C@Cu_1.04_	H_2_SO_4_/N_2_	0.495	15.1	26.49	0.2	339.97	0.7	0.5927
	KHCO_3_ N_2_	0.041	68.9	143.19	12.4	135.07	562	1.319
	KHCO_3_/CO_2_	0.265	73.7	8.52	0.8	32.02	7.9	1.3357
PL6C@Cu_1.24_	H_2_SO_4_/N_2_	0.487	14.6	132.40	382.0	121.04	71.1	0.0019
	KHCO_3_/N_2_	0.170	73.0	67.25	139.9	15.13	113.3	0.0010
	KHCO_3_/CO_2_	0.320	63.6	12.90	7.4	22.77	39.2	0.1903

a All solutions were prepared with a concentration of 0.1 mol L^–1^.

Finally, to estimate the electrochemically active surface area (ECSA), the double-layer capacitance was determined via cyclic voltammetry at varying scan rates. Figure 5d shows the variation of current density (*j*) as a function of the scan rate obtained for the different PL6C@Cu_*x*_ composites, from which the interfacial capacitance of each material was determined. Based on the slope of the curves obtained, the PL6C@Cu_1.04_ composite exhibited the highest specific capacitance, with a value of 280.83 μF·cm^–2^, indicating a larger electrochemically active surface area and better accessibility to catalytic sites. In comparison, the other materials showed decreasing C_dl_ values: PL6C@Cu_1.24_ (168.50 μF·cm^–2^), PL6C@Cu_0.48_ (141.97 μF·cm^–2^), PL6C@Cu_0.11_ (137.61 μF·cm^–2^), and bare PL6C (122.17 μF·cm^–2^). The ECSA was estimated, yielding values of 3.49, 3.93, 4.05, 8.02, and 4.81 for the bare PL6C, PL6C@Cu_0.11_, PL6C@Cu_0.48_, PL6C@Cu_1.04_, and PL6C@Cu_1.24_ samples, respectively. These results indicate that the incorporation of copper into PL6C can significantly enhance the surface electrochemical activity, whereas excessive metal loading may lead to active site blocking or particle agglomeration, thus reducing the effective surface area available for the reaction.

The copper-specific surface area (SSA_Cu_) followed a nonmonotonic, volcano-type relationship as a function of metal loading. At the lowest loading (0.11 wt.%), we obtained a high SSA_Cu_ of 501.3 m^2^ g_Cu_^–1^ showing good initial dispersion of the copper phase. Interestingly, the SSA_Cu_ decreased to 145.7 m^2^ g_Cu_^–1^ for the PL6C@Cu_0.48_ sample, which may suggest the onset of initial cluster formation or a localized growth stage. However, the specific area reached its maximum of 544.8 m^2^ g_Cu_^–1^ for the PL6C@Cu_1.04_ catalyst, indicating that this specific loading provides the optimal density of accessible active sites within the carbon framework. Conversely, a further increase in copper content to 1.24 wt.% led to a dramatic decrease in the normalized surface area, dropping to 133.0 m^2^ g_Cu_^–1^. This 4-fold reduction in SSA_Cu_ compared to the optimized sample correlates with the particle agglomeration seen in TEM and explains the mesopore blockage that hinders CO_2_ accessibility and catalytic performance.

The aromatic character of the PL6C support, associated with its *sp* ^*2*^ hybridized carbon domains, is fundamental for ensuring high electrical conductivity and providing anchoring sites for the stabilization of Cu nanoparticles.^39^ The volcano-type relationship observed as a function of Cu content suggests that the catalytic performance is governed by a synergy between the metal and the support. The optimized Cu loading of 1.04 wt % appears to provide a favorable density of accessible Cu sites while preserving a suitable interfacial microenvironment for CO_2_ reduction. In contrast, lower or higher Cu loadings may result in less favorable Cu distribution and reduced accessibility of active sites, as suggested by the TEM analysis shown in Figure S2. This is particularly relevant for PL6C@Cu_1.24_, which exhibits a higher Cu content and slightly higher estimated theoretical Cu surface area, but does not show the best CO_2_RR response. Therefore, the lower activity observed for the nonoptimized samples cannot be explained solely by the total Cu amount, but is likely related to differences in Cu dispersion, active-site accessibility, and local CO_2_ availability at the electrode interface.

### 3.3 Catalytic Activity and Selectivity toward CO_2_ Electro-Reduction

The catalytic activity of the materials was evaluated using linear sweep voltammetry (LSV). Figure 6a shows the polarization curves; the detailed onset potential values obtained for all the composites under N_2_- and CO_2_-saturated atmospheres are summarized in Table S2. The bare PL6C exhibited no significant variation in onset potentials between the N_2_- and CO_2_-saturated electrolytes (−0.70 and −0.72 V vs RHE, respectively); this is clearly indicative of the absence of specific active sites for CO_2_ activation. In contrast, the incorporation of copper into PL6C promoted a pronounced anodic shift in the onset potential under CO_2_ atmosphere. As detailed in Table S2, this effect is strongly dependent on metal loading, with the onset potential shifting from −0.70 V vs RHE for PL6C@Cu_0.11_ to an optimum value of −0.53 V vs RHE for the PL6C@Cu_1.04_ composite.

Among the materials investigated, the PL6C@Cu_1.04_ exhibited the largest positive shift relative to its N_2_ baseline (ΔE ∼ 110 mV); in essence, this shows that it exhibited the most favorable kinetics for CO_2_ reduction. This superior performance can be attributed to an optimal balance between metal loading and surface dispersion. To further support this interpretation, the theoretical Cu surface area was estimated from the Cu loading and average particle size, assuming spherical Cu particles (Table S3, see Supporting Information). Although PL6C@Cu_1.24_ presented a slightly higher estimated Cu surface area than PL6C@Cu_1.04_, it did not show the best CO_2_RR response. This result suggests that the CO_2_RR activity is not governed only by the total amount of Cu or by the estimated Cu surface area, but also by the distribution, accessibility, and interfacial environment of the Cu sites. While lower loadings (PL6C@Cu_0.11_ and PL6C@Cu_0.48_) provide fewer active sites, the PL6C@Cu_1.24_ sample exhibits localized agglomeration into clusters (Figure S2a, see Supporting Information), which may reduce the effective electrochemical surface area and lead to the observed regression of the onset potential to −0.60 V. In contrast, the PL6C@Cu_1.04_ catalyst exhibits an average particle size of 8.76 nm, a size range that, according to the literature,^31^ favors the density of low-coordinated sites for CO production. This behavior, which is consistent with the impedance and capacitance results, shows that excessive metal loading may promote particle agglomeration, thereby reducing the intrinsic catalytic activity.

To deconvolute the total faradaic current and identify gaseous products generated at the electrode/electrolyte interface, RRDE voltammetry analysis was carried out. Instead of the conventional amperometric mode (fixed ring potential), a ring voltammetric detection mode was used, as described by Zhang et al. (2020).^18,40^ This approach enables the qualitative “fingerprinting” of reaction products based on their characteristic oxidation potentials on the Pt ring electrode. Figure 6b schematically illustrates the ring-collection experimental setup. While the disk potential was held constant at a value sufficient to drive reduction, the ring potential was scanned linearly.

To identify the intermediates generated during the reaction, the electrochemical fingerprints of potential byproducts were determined using ring voltammetry. The stability of the PL6C support was verified through control measurements in N_2_-saturated electrolyte, Figure S5 (see Supporting Information). The absence of oxidation peaks in the ring during these experiments indicates that the CO detected during CO_2_RR originates from the reduction of the gaseous reactant rather than the electrochemical oxidation or degradation of the carbon matrix. Figure 6c shows the cyclic voltammograms obtained for standard solutions of formate (HCOO^–^) and methanol (CH_3_OH), along with the KHCO_3_ electrolyte baseline. The CO oxidation profile, obtained separately to avoid cross-contamination, is shown in Figure S6. The electrolyte background (black curve in Figure 6c) exhibits no significant oxidation features. In aqueous media, the dissolved CO_2_ establishes a chemical equilibrium described by eqs 7–10. Although this leads to local pH changes due to the release of H^+^ ions,^11,41,42^ the carbonate species themselves do not undergo electrochemical oxidation on the Pt ring within the studied potential window.

7 $${\mathrm{CO}}_{2}\left(g\right)⇌{\mathrm{CO}}_{2}\left(\mathrm{aq}\right)$$

8 $${\mathrm{CO}}_{2}\left(\mathrm{aq}\right){+H}_{2}O⇌H_{2}{\mathrm{CO}}_{3}$$

9 $$H_{2}{\mathrm{CO}}_{3}⇌H^{+}{{+\mathrm{HCO}}_{3}}^{-}$$

10 $${{\mathrm{HCO}}_{3}}^{-}⇌H^{+}{{+\mathrm{CO}}_{3}}^{\mathrm{2-}}$$

The detection methodology employed here mirrors the established approach for H_2_O_2_ monitoring in oxygen reduction reaction (ORR).^43^ It should be noted, however, that instead of a fixed potential, the ring electrode was scanned from 0.06 to 1.40 V vs RHE. This wide window allows the discrimination of products based on their specific oxidation potentials: while CO typically oxidizes at 0.8 V (Figure S6, see Supporting Information), formate and methanol exhibit distinct oxidation onset potentials, as observed in Figure 6c. These diagnostic baselines were subsequently used to identify the species generated on the PL6C@Cu_*x*_ modified disk under cathodic polarization (−0.2 to −1.2 V vs RHE) and transported by convective forces to the ring.

After establishing the calibration profiles, the product distributions generated by the catalysts were investigated. Figure 6d shows the series of voltammograms recorded on the Pt ring (scanning from 0.06 to 1.40 V vs RHE) while the disk electrode, modified with PL6C@Cu_1.04_, was held at constant cathodic potentials ranging from −0.20 V to −1.25 V vs RHE in CO_2_-saturated electrolyte. At low overpotentials (E_disk_ > −0.6 V vs RHE), the ring response remains confined to the capacitive baseline, indicating negligible product formation. However, as the disk potential is shifted to more negative values (−0.8 V to −1.25 V vs RHE), a distinct anodic feature emerges and progressively intensifies. Specifically, a broad oxidation peak centered at approximately 0.8 V vs RHE is observed. By cross-referencing this profile with the diagnostic fingerprints established in Figure S6, this feature is unambiguously assigned to the surface oxidation of CO—see eq 11.^6,42^

11 $${\mathrm{CO}}_{\mathrm{ads}}{+H}_{2}O\to {\mathrm{CO}}_{2}{+2H}^{+}{+2e}^{-}$$

The ring current density (*j*_*ring*_) increases proportionally with the cathodic polarization of the disk, confirming that the rate of CO production on the PL6C@Cu_1.04_ surface is potential-dependent. Furthermore, the absence of significant oxidation currents in the potential regions characteristic of formate or methanol (typically <0.6 V vs RHE, as shown in Figure 6c) suggests that, under these hydrodynamic conditions, CO is the predominant detectable product. This highlights the selectivity of the Cu nanoparticles supported on PL6C toward the CO formation pathway.^44^

To verify that CO evolution originates exclusively from the Cu active sites and to assess the impact of metal loading on the production rate, the same diagnostic methodology was applied to the remaining catalysts (Figure S7, see Supporting Information). The bare PL6C catalyst (Figure S7a, see Supporting Information) showed no evidence of oxidation features within the monitored ring potential window; this essentially confirms that the carbonaceous matrix is inactive when it comes to the conversion of CO_2_ to CO and favors HER primarily. In contrast, the other Cu-modified catalysts (Figure S7b–d) displayed the characteristic CO oxidation peaks, confirming the intrinsic activity of the Cu nanoparticles, regardless of the metal loading. However, a quantitative analysis reveals a crucial distinction: while active, these materials exhibited j_ring_ consistently lower than that recorded by the PL6C@Cu_1.04_. This observation shows that the PL6C@Cu_1.04_ sample exhibits an optimal balance between active site dispersion and electrochemical accessibility, maximizing product generation per geometric area compared to both lower loadings (limited by a scarcity of active sites) and higher loading −1.24 (limited by agglomeration effects).

To evaluate the interfacial CO production during the initial seconds of the experiment, the currents recorded at the ring were used to estimate the apparent partial current density for CO (*j*_*CO*_), accounting for the collection efficiency (N = 0.37). This approach provides a semiquantitative assessment of the catalyst’s selectivity by detecting products as they are generated at the disk-electrolyte interface.^45^ The ring current values and the respective *j*_*CO*_ for all catalysts at a disk potential of −1.1 V vs RHE are summarized in Table S4 (see Supporting Information). According to Table S4, while the bare PL6C support exhibited a low j_CO_ of 0.10 ± 0.003 mA cm^2^, copper incorporation significantly enhanced CO evolution within the first seconds of the reaction. Specifically, the optimized PL6C@Cu_1.04_ catalyst demonstrated a nearly 4-fold increase in activity compared to the support, reaching a j_CO_ of 0.38 ± 0.011 mA cm^2^ at −1.1 V vs RHE. Although the highest current density was recorded for the PL6C@Cu_1.24_ sample (0.65 ± 0.019 mA cm^2^), the efficiency gain per metal mass begins to plateau, reinforcing the importance of the optimized dispersion found at lower loadings.

To compare the performance of the PL6C@Cu_1.04_ catalyst, its activity was compared with systems previously reported in the literature. While noble metal catalysts, such as Au and Ag, typically exhibit CO onset potentials in the range −0.4 V to −0.7 V vs RHE (depending on the support and electrolyte),^46,47^ the catalyst with 1.04 wt.% demonstrates a competitive reaction onset at −0.53 V vs RHE.

### 3.4 Interfacial Environment and Local pH Modulation

To elucidate the causes behind the enhancement of catalytic activity, the local CO_2_ environment on the catalyst surface was probed by cyclic voltammetry using 1,4-benzoquinone (BQ) as a redox probe, since this molecule can reversibly bind to CO_2_,^48–50^ Figure S8 (see Supporting Information). The anodic potential shifts (ΔE), as well as the corresponding local CO_2_ concentrations estimated using the Nernst equation for a chemically equilibrated system, are summarized in Table S5 (see Supporting Information). These potential shifts reflect the coupled influence of local CO_2_ concentration and interfacial pH changes, as both parameters are intrinsically linked through the CO_2_/HCO_3_^–^ equilibrium.

The data shows that the bare PL6C and the low metal-loading sample (PL6C@Cu_0.11_) exhibited identical potential shifts (ΔE = 0.105 V). This suggests that at low copper loadings, the interfacial affinity toward CO_2_ may be predominantly governed by the mesoporous carbon architecture. In contrast, a pronounced deviation is observed at optimal metal loadings. The PL6C@Cu_1.04_ catalyst exhibited the highest anodic shift (0.140 V vs Ag/AgCl), which is consistent with an increased local CO_2_ availability compared to both the carbon support and the other composites.

This apparent increase in reactant availability is in good agreement with the superior catalytic activity observed in the LSV measurements; clearly, it points to a possible synergistic effect between copper incorporation and the porous carbon framework in promoting modified interfacial CO_2_ enrichment. The subsequent decrease in ΔE recorded in the PL6C@Cu_1.24_ sample (0.130 V vs Ag/AgCl) suggests that excessive metal loading may lead to partial pore blockage or particle agglomeration, slightly diminishing the effective CO_2_ “reservoir effect”. Thus, the observed ΔE serves as a quantitative proxy for the overall interfacial environment enhancement.

This correlation indicates that the superior performance of the PL6C@Cu_1.04_ sample is not driven solely by electronic effects, but also by an optimized interfacial environment that actively concentrates CO_2_ near the active sites, thereby overcoming mass transport limitations and promoting the kinetics of CO evolution or C–C coupling.^12,22,51^

The local pH modulation is governed by the Cu loading and its impact on CO_2_RR kinetics. Higher copper nanoparticle loadings (up to 1.04 wt.%) increase the active site density, promoting a greater flux of OH^–^ ions at the interface. Due to the confined mesoporous environment of the PL6C support, these ions accumulate locally rather than diffusing rapidly into the bulk electrolyte, leading to a significant increase in local pH, as suggested by the BQ redox shifts. This interfacial alkalinity is self-regulated by the catalyst activity; notably, the PL6C@Cu_1.24_ sample exhibited a smaller pH shift than the 1.04 wt.% analog. This is attributed to particle agglomeration and pore blockage, which decrease the accessible surface area and, consequently, reduce the local OH^–^ generation rate. These observations are consistent with reports suggesting that the interfacial microenvironment is sensitive to both the reaction rate and the morphological confinement of the electrode.^38,52,53^

## 4 Conclusion

The present work showed that copper loading plays an important role in tuning the electrocatalytic response of PL6C-supported catalysts for the CO_2_ reduction reaction. Among the synthesized materials, the PL6C@Cu_1.04_ showed the most favorable catalytic, exhibiting a more positive onset potential of −0.53 V vs RHE and selectivity toward CO evolution, as indicated by the RRDE measurements. Crucially, the mechanistic investigation conducted using a quinone redox probe provided insights into the interfacial behavior of these catalysts. It was found that the enhanced performance exhibited by the PL6C@Cu_1.04_ catalyst was driven by a substantial local CO_2_ enrichment on the electrode surface, evidenced by a remarkable anodic shift of 0.140 V. However, this behavior cannot be attributed solely to the increase in Cu content or theoretical Cu surface area, since the catalyst with the highest Cu loading did not provide the best overall CO_2_RR response. These findings suggest that the optimized Cu dispersion on the mesoporous support contributes to a reservoir effect, increasing local reactant availability favoring the CO_2_ reduction pathway. Overall, these results highlight the importance of modulating the interfacial microenvironment for designing next-generation CO_2_RR catalysts.

## Supplementary Material



## Notes

Additional details on experimental procedures; structural and morphological characterization; electrochemical performance of the catalysts (PDF)
