# Supplementary material for: Interfacial Insights into the Efficient Electroconversion of CO2 to CO Using Cu-Based Catalysts Supported on Printex L6 Carbon
Source: ACS Omega. 2026 Aug 4;11(32):47626–37. doi: 10.1021/acsomega.6c02410 (PMC13491461; doi:10.1021/acsomega.6c02410)
Supplement: Supplementary file 1 [file ao6c02410_si_001.pdf]

Supporting Information for

Interfacial insights into the efficient electroconversion of CO_2 to CO using Cu-based catalysts supported on Printex L6 carbon

Thalita F. da Silva [*a], *Larissa T. Cardoso* [a], *Marciélli K. R. de Souza* [a], *Julio César Lourenço*[a], *Guilherme V. Fortunato* [b], *and Marcos R. V. Lanza* [*a]

[a] São Carlos Institute of Chemistry, University of São Paulo, Trab. São Carlense Avenue, 400, São Carlos, SP, Brazil.

[b] Sustainable Energy Materials, Technical University Munich, Campus Straubing, Schulgasse 22, Straubing 94315, Germany.

*Corresponding author's e-mail address:

Thalita Ferreira da Silva thalitafs@usp.br

Marcos Roberto de Vasconcelos Lanza marcoslanza@usp.br

1. Polarographic determination of copper in PL6C

A methodology was developed to determine the copper content in the PL6C@Cu_x catalysts. The electrolyte consisted of an acetate buffer (pH 4.99), prepared from acetic acid and sodium acetate at 0.1 mol L^{-1} each, mixed in a 1:4 (v/v) ratio. The samples were subjected to an acid digestion process as follows: 10 mg of each material was treated with 1 mL of concentrated nitric acid and heated for 10 min. Subsequently, 0.5 mL of H_2O_2 was added to complete the digestion. After completion of the reaction, aliquots of 300 µL of the digestate were diluted to 15 mL with acetate buffer prior to analysis. The copper concentration was determined using the successive standard addition method, in which five consecutive additions of 10 µL of a copper standard solution were performed. Polarographic measurements were carried out using a Metrohm polarograph (µAutolab type III, Autolab IME 663), equipped with a 663 VA Stand cell, an Ag/AgCl reference electrode, and a graphite carbon rod as the counter electrode. Current responses as a function of potential were obtained by differential pulse polarography (SMDE) over a potential range of -0.40 to 0.20 V, with a potential step of 0.005 V, a modulation amplitude of 0.05 V, a modulation time of 0.04 s, and a time interval of 0.10 s.

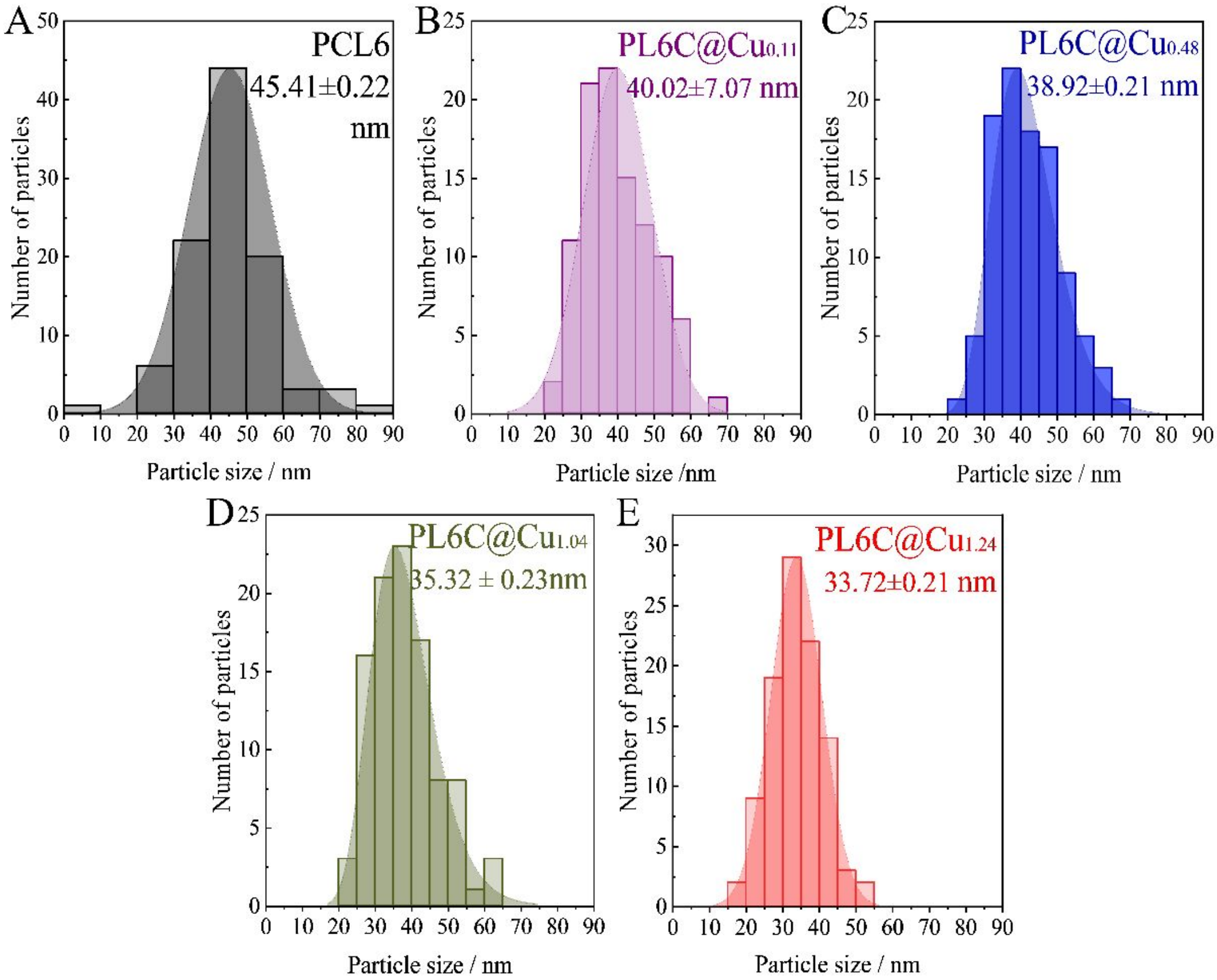

Figure S1. Particle size distribution of the (a) PL6C, (b) PL6C@$Cu_{0.11}$, (c) PL6C@$Cu_{0.48}$, (d) PL6C@$Cu_{1.04}$, and (e) PL6C@$Cu_{1.24}$. Each distribution plot is based on measurements of 100 particles obtained from field emission scanning electron microscopy (FE-SEM) analysis. Particle size is defined as the average of the lengths of a particle.

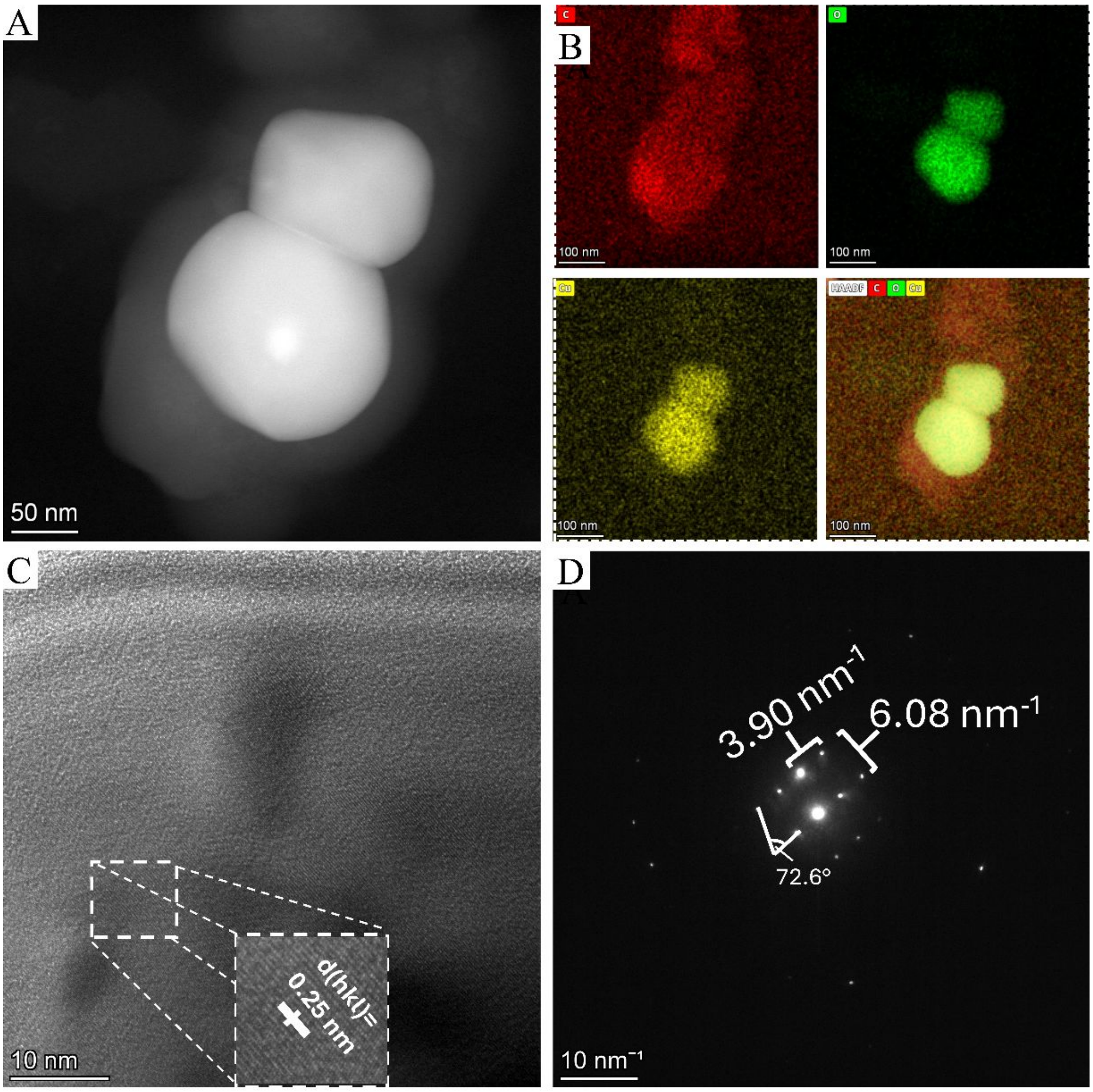

Figure S2. Detailed characterization of the PL6C@$Cu_{1.24}$ catalyst: (a) STEM-HAADF image of a Cu agglomerate, (b) corresponding EDS elemental mapping, (c) HR-TEM image highlighting the lattice d-spacing, and (d) nanodiffraction pattern of the selected area.

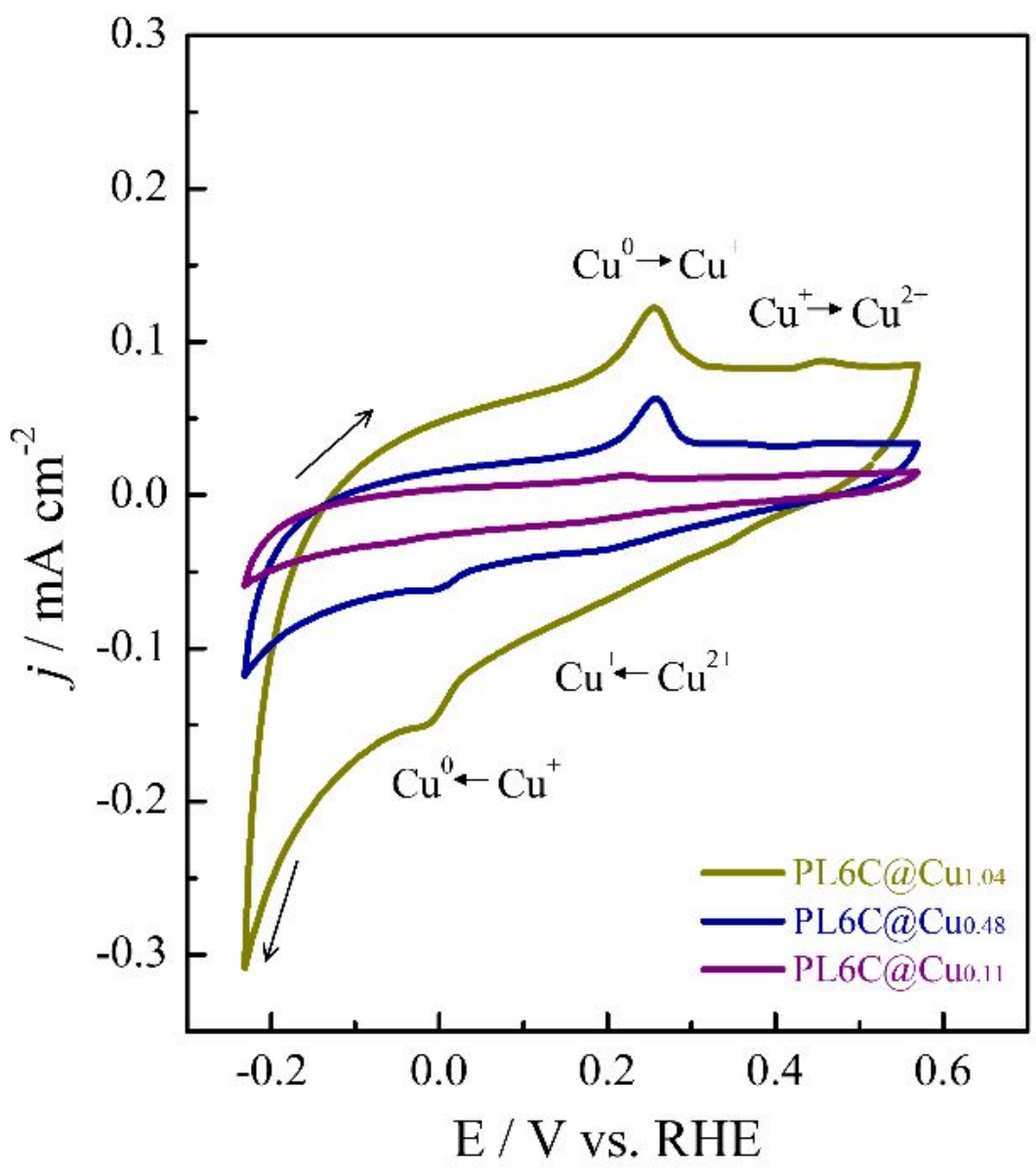

Figure S3. Cyclic voltammograms of the catalysts (PL6C@Cu_x) were recorded in 0.1 M H_2SO_4 saturated with N_2, using a scan rate of 50 mV·s^{-1}.

Table S1. Structural disorder assessment via D/G band analysis in carbon catalysts.

**Catalysts**	**Band / cm^{-1}**		**I_D/I_G**
	**D**	**G**	
PL6C	1318	1586	1.36
PL6C@$Cu_{0.11}$	1340	1596	1.34
PL6C@$Cu_{0.48}$	1337	1595	1.33
PL6C@$Cu_{1.04}$	1345	1592	1.40
PL6C@$Cu_{1.24}$	1343	1600	1.25

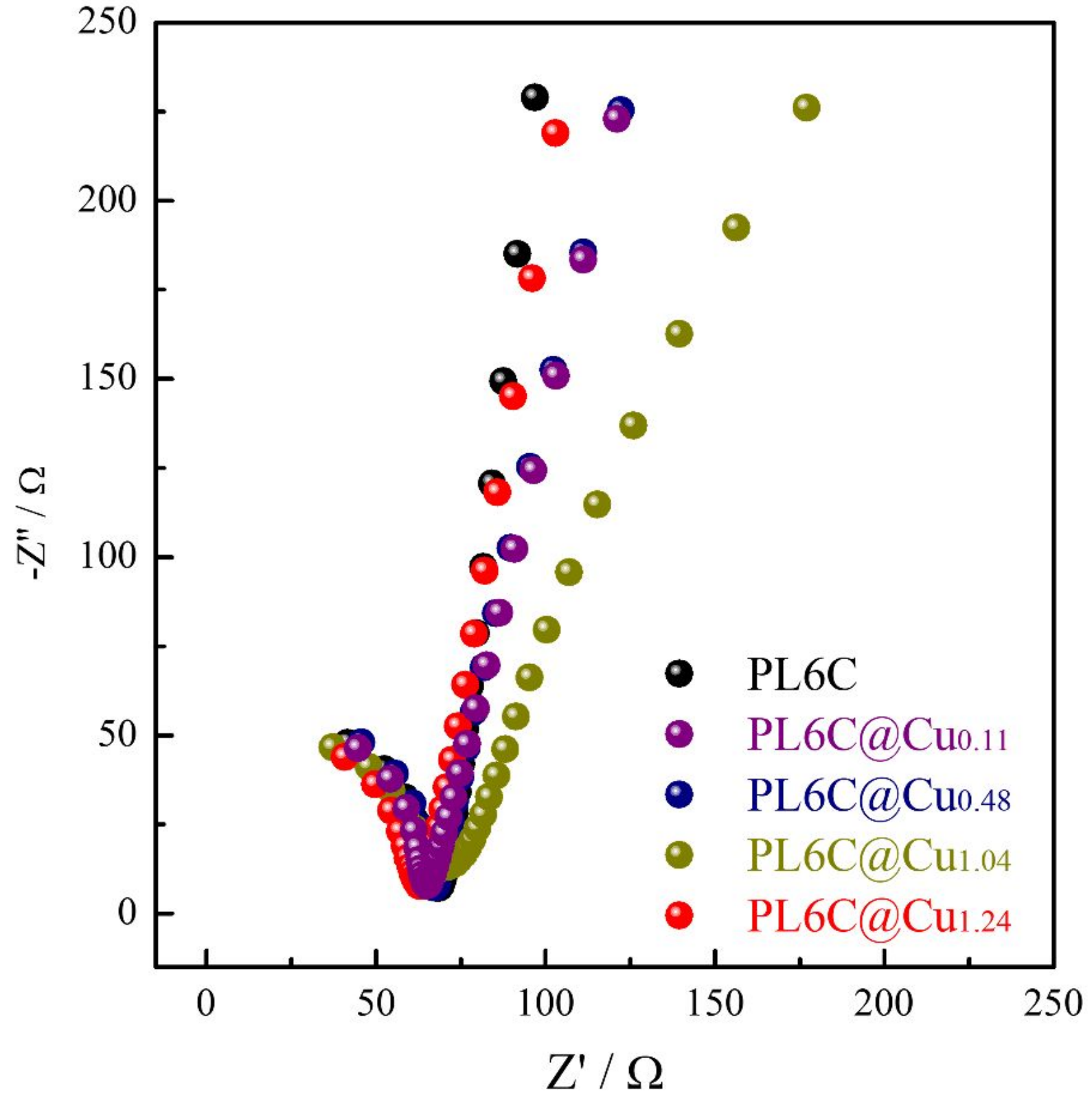

Figure S4. Magnification of the Nyquist plot for the catalysts.

Table S2. Onset potentials (E_{onset}) for the electrocatalysts under N_2- and CO_2-saturated conditions at 1600 rpm and a scan rate of 5 mV·s^{-1}.

Saturation	Electrocatalyst	E_{onset} vs. RHE
N_2	PL6C	-0.70
	PL6C@$Cu_{0.11}$	-0.93
	PL6C@$Cu_{0.48}$	-0.70
	PL6C@$Cu_{1.04}$	-0.64
	PL6C@$Cu_{1.24}$	-0.65
CO_2	PL6C	-0.72
	PL6C@$Cu_{0.11}$	-0.70
	PL6C@$Cu_{0.48}$	-0.56
	PL6C@$Cu_{1.04}$	-0.53
	PL6C@$Cu_{1.24}$	-0.60

Table S3. Estimated theoretical Cu surface area of PL6C@Cu catalysts based on Cu loading and average particle size.

Catalyst	Cu loading / wt%	Particle size / nm	*Theoretical Cu surface rea / cm^2	**Cu area normalized by geo area / $cm^2Cu\ cm^{-2}$ geo
PL6C@$Cu_{0.11}$	0.11	8.40	0.017	0.070
PL6C@$Cu_{0.48}$	0.48	10.22	0.062	0.252
PL6C@$Cu_{1.04}$	1.04	8.76	0.157	0.636
PL6C@$Cu_{1.24}$	1.24	9.00	0.183	0.738

Notes: *The theoretical Cu surface area was estimated assuming spherical Cu particles according to: $A_{\mathrm{Cu,theo}} = \frac{6m_{\mathrm{Cu}}}{\rho_{\mathrm{Cu}}d}$, where $m_{\mathrm{Cu}}$is the mass of Cu deposited on the electrode, $\rho_{\mathrm{Cu}}$is the density of Cu (8.96 g cm^{-3}), and $d$is the average particle diameter. **The Cu surface area normalized by the geometric electrode area was calculated as:$A_{\mathrm{Cu,norm}} = \frac{A_{\mathrm{Cu,theo}}}{A_{\mathrm{geo}}}$.

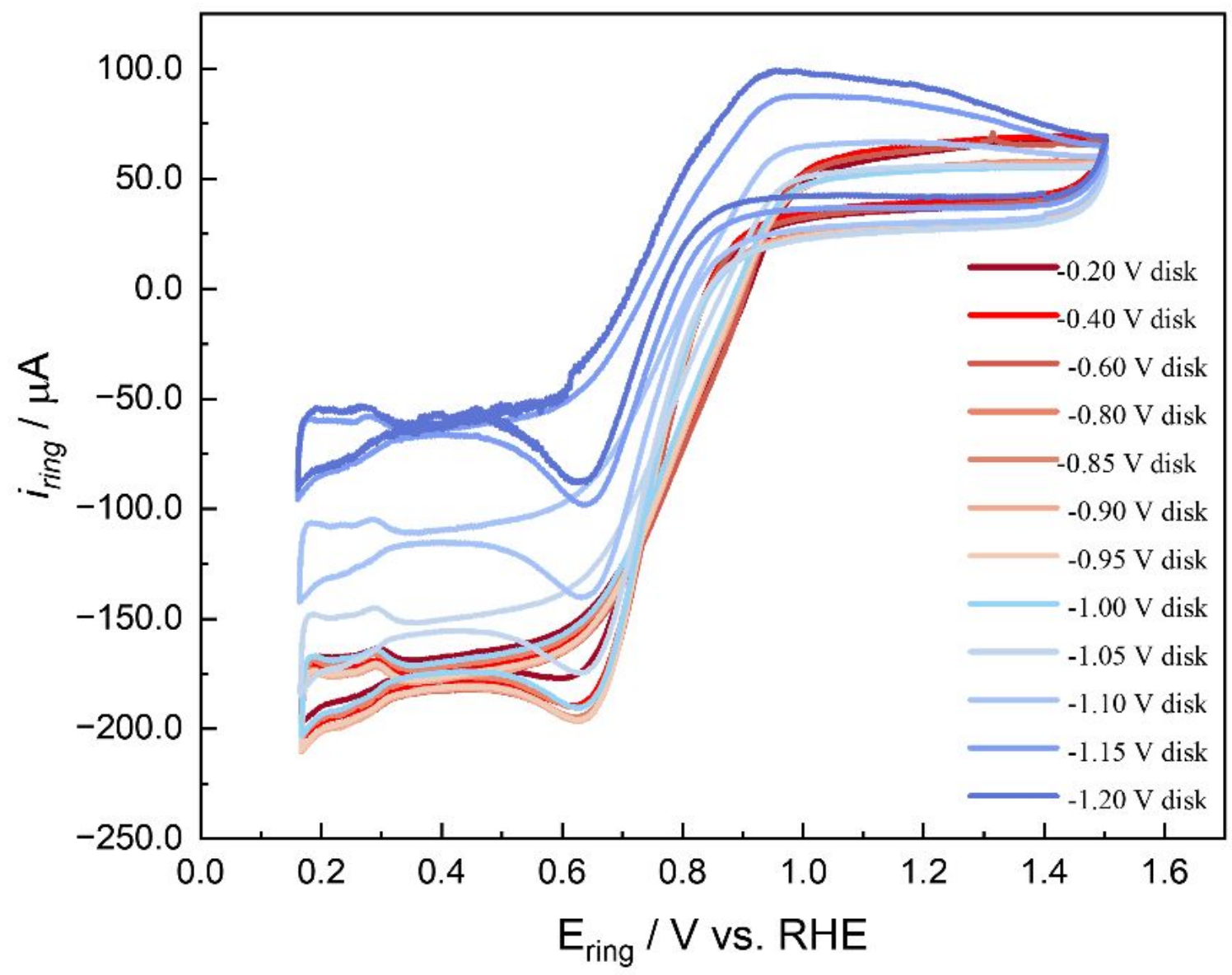

Figure S5. Cyclic voltammograms (CVs) obtained at the Pt ring electrode at different constant disk potentials for the bare PL6C support in N_2-saturated electrolyte.

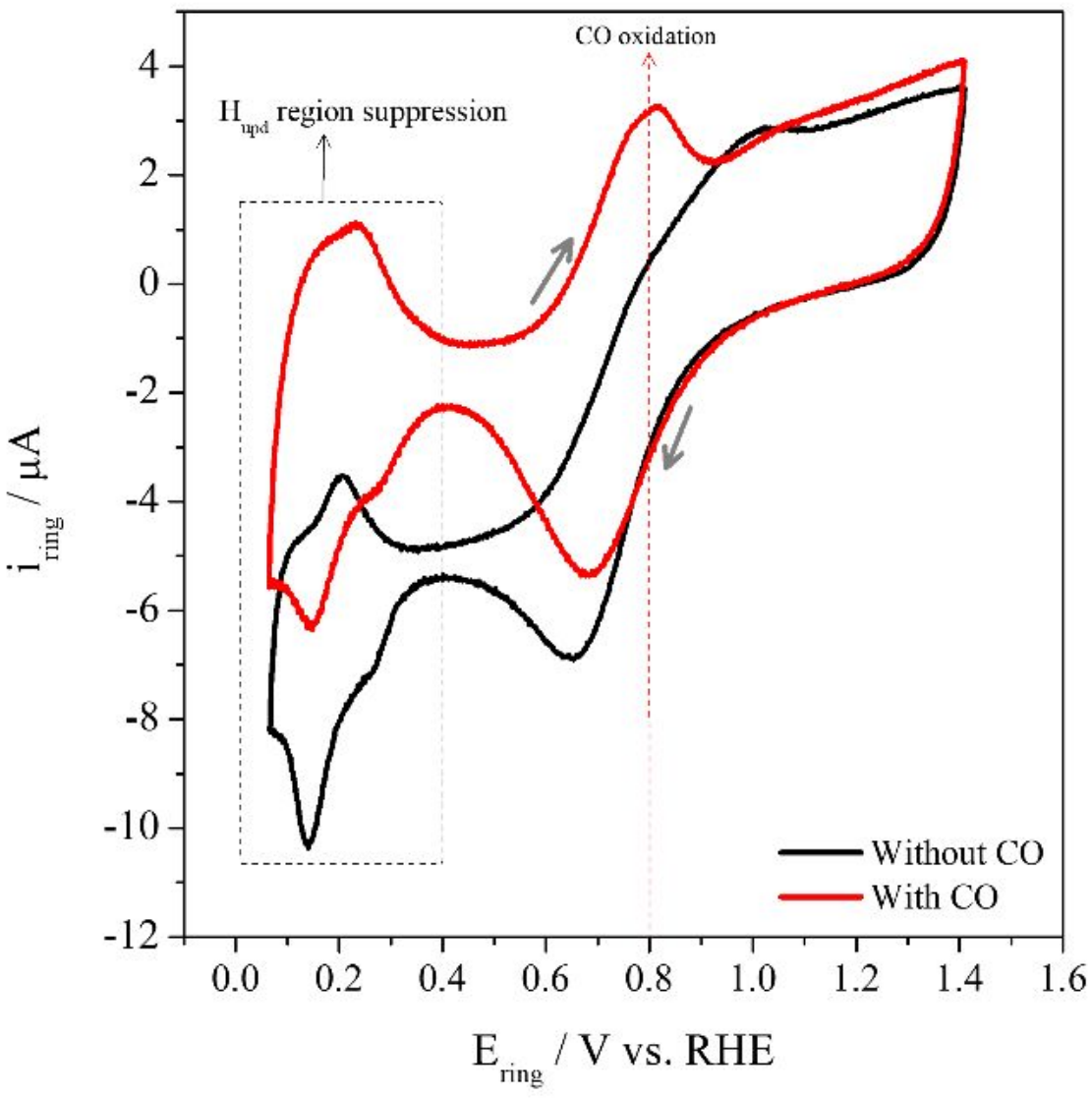

Figure S6. Oxidation of CO and $KHCO_3$ at the platinum ring electrode at 1600 rpm, with a scan rate of 100 mV·s^{-1}.

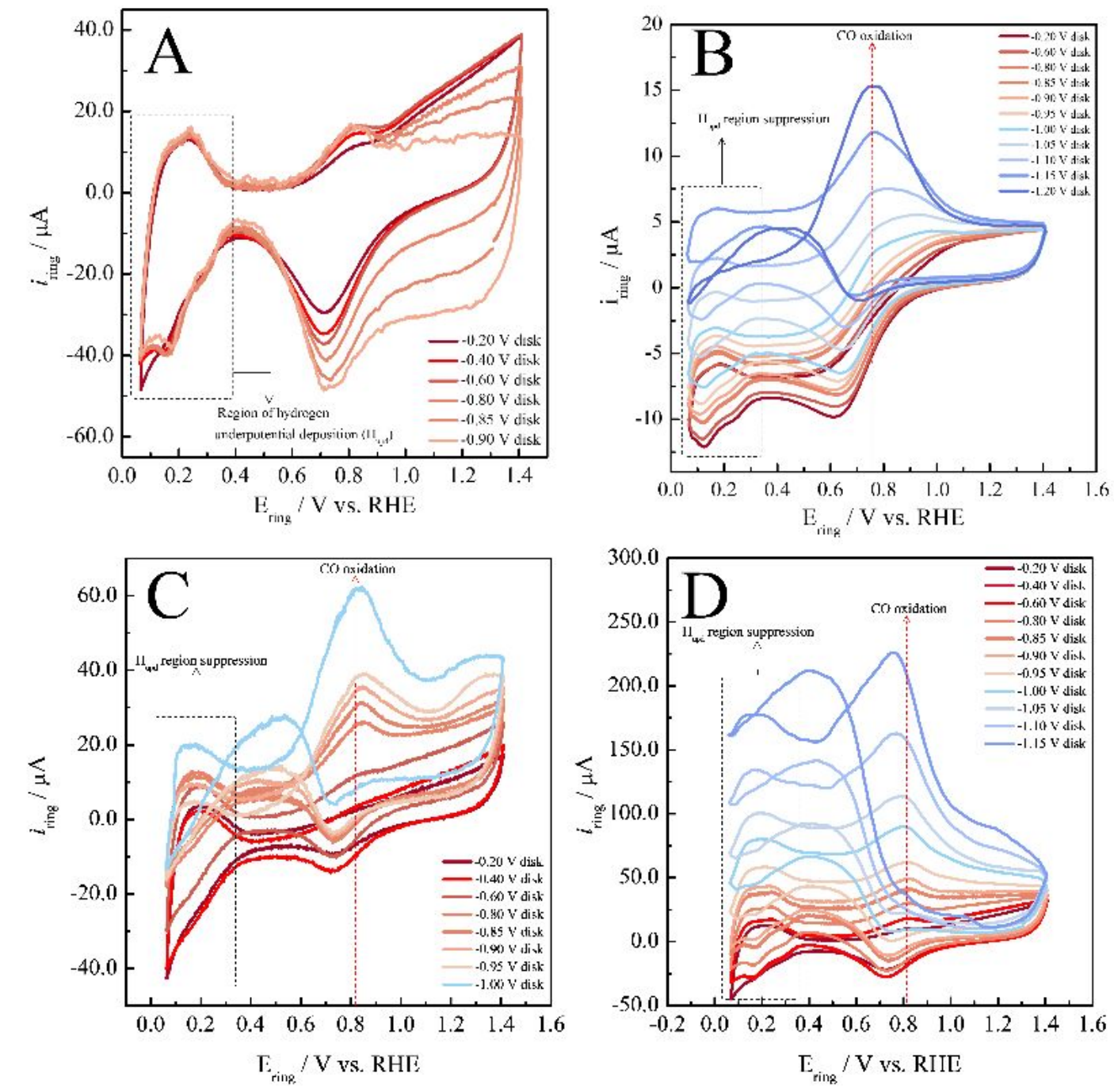

Figure S7. Cyclic voltammograms (CVs) obtained at the Pt ring electrode are shown for different constant potentials applied to the glassy carbon disk electrode modified with the synthesized catalysts: (a) PL6C; (b) PL6C@$Cu_{0.11}$; (c) PL6C@$Cu_{0.48}$; and (d) PL6C@$Cu_{1.24}$.

Table S4. Electrochemical parameters and estimated apparent CO partial current densities ($j_{CO,\ app}$) for all catalysts at a disk potential of -1.1 V vs. RHE.

**Samples**	**I_{ring} /μA**	**$j_{CO,\ app}$ / mA cm^{-2}**	**Cu mass on disk / μg**	**Mass activity / A g$^{-1}{}_{Cu}$**
PL6C	9.23 ±0.28	0.101 ± 0.003	-	-
PL6C@$Cu_{0.11}$	3.39 ±0.10	0.037 ±0.001	0.0218	420.7 ±12.6
PL6C@$Cu_{0.48}$	31.16 ± 0.93	0.340 ± 0.010	0.0950	886.1 ±26.6
PL6C@$Cu_{1.04}$	34.57 ±1.04	0.377 ±0.011	0.2059	453.7 ±13.6
PL6C@$Cu_{1.24}$	59.33 ±1.78	0.648 ±0.019	0.2455	653.1 ±19.6

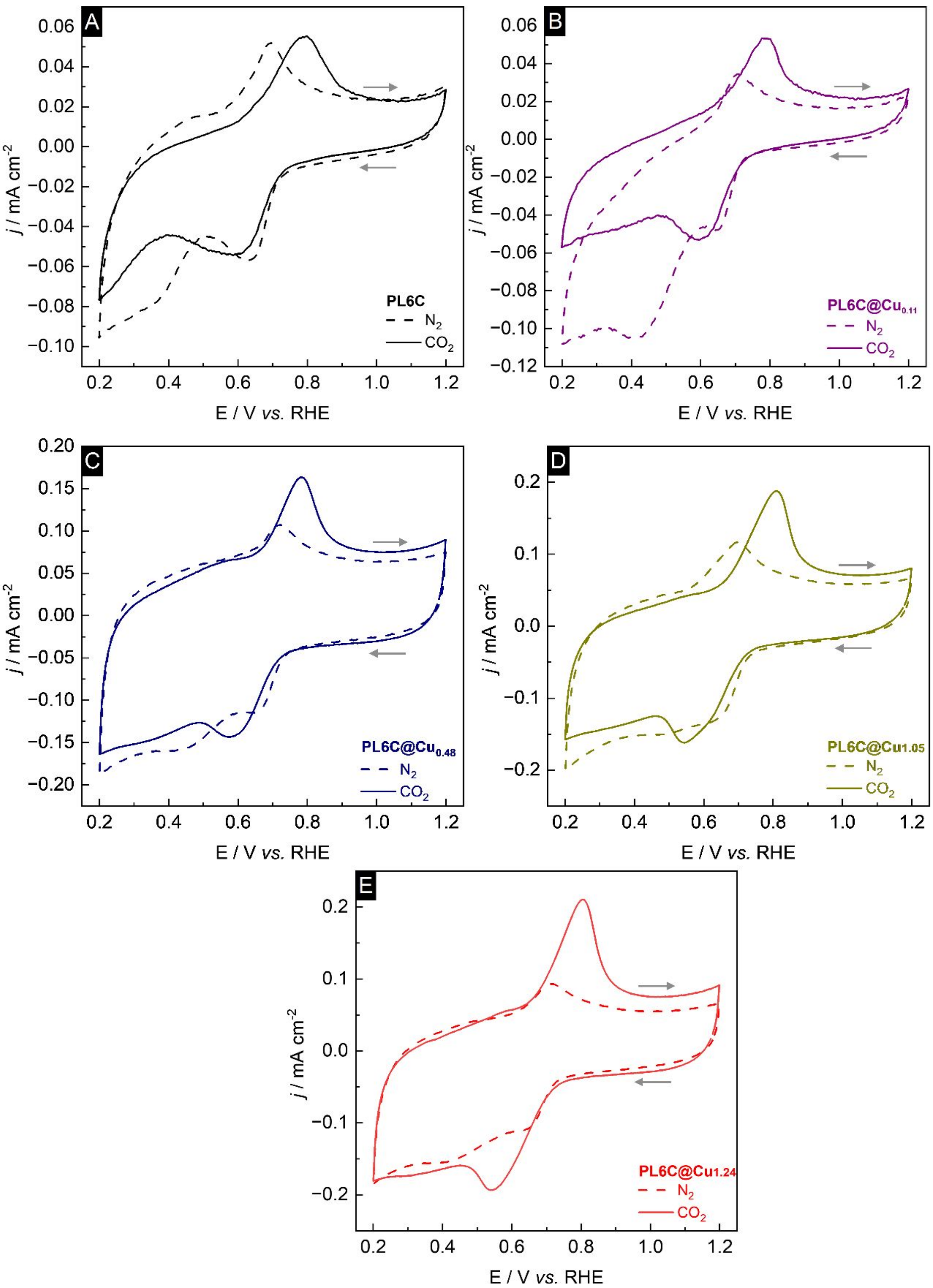

Figure S8. Electrochemical response of a 1.0 mM 1,4-benzoquinone (BQ) probe on PL6C and PL6C@Cu_x catalysts: (a) PL6C, (b) PL6C@$Cu_{0.11}$, (c) PL6C@$Cu_{0.48}$, (d) PL6C@$Cu_{1.04}$, and (e) PL6C@$Cu_{1.24}$. Measurements were obtained in 0.1 M $KHCO_3$ at 50 mV s^{-1} in N_2-purged (dashed) and CO_2-saturated (solid) electrolytes.

Table S5. Voltammetric parameters extracted from the redox probe analysis.

	ΔE/ mV	Relative CO_2 Affinity[a]
PL6C	105	1
PL6C@$Cu_{0.11}$	105	1
PL6C@$Cu_{0.48}$	100	0.7
PL6C@$Cu_{1.04}$	140	15.2
PL6C@$Cu_{1.24}$	130	7

[a] Calculated as Relative CO_2 affinity $=10^{\frac{(\Delta(\Delta E)}{S}}$, where $\Delta(\Delta E)$ is the difference in potential shift between the sample and the PL6C support, and S is the Nernstian slope (approx. 29.6 mV dec^{-1} at 298 K for a 2-electron process).
